# 

**Published:** 1887-01

**Authors:** 


					﻿VOL VIL	JANUARY, 1887.	0	No. 1.
THE
pOMCEDPATHlC pHY^lClAJl,
A MONTHLY JOURNAL OF MEDICAL SCIENCE.
“ He is a freeman whom truth makes free, and all are slaves beside.”
“ Seek the Truth: come whence it may, cost what it wiU.”
EDITED BY
Edmund j. lee, me. d.,
AND
WALTER M. JAMES, M. D.
PHILADELPHIA:
No. 1123 SPRUCE STREET.
TST3CW YORK,:
A. L. CHATTERTON & COMPANY, Publisher’s Agents.
LONDON":
ALFRED HEATH & CO., Sole Agents eor Great Britain,
114 Ebury Street, Eaton Square.
Yearly Subscription, $2.50, invariably in advance. Single numbers, 25 cts.
Entered at the Philadelphia Peet-Office aa Second-Class Mall Matter.
				

